# Eye image effect in the context of pedestrian safety: a French questionnaire study

**DOI:** 10.12688/f1000research.76062.1

**Published:** 2022-02-23

**Authors:** Cédric Sueur, Anthony Piermattéo, Marie Pelé

**Affiliations:** 1 Institut Universitaire de France, Paris, China; 2 IPHC, UMR7178, Université de Strasbourg, CNRS, Strasbourg, France; 3 ETHICS EA7446, Lille Catholic University, Lille, France

**Keywords:** prosociality, road crossing, reputation, accident prevention, pedestrian behavior

## Abstract

Human behavior is influenced by the presence of others, which scientists also call ‘the audience effect’. The use of social control to produce more cooperative behaviors may positively influence road use and safety. This study uses an online questionnaire to test how eyes images affect the behavior of pedestrians when crossing a road. Different eyes images of men, women and a child with different facial expressions -neutral, friendly and angry- were presented to participants who were asked what they would feel by looking at these images before crossing a signalized road. Participants completed a questionnaire of 20 questions about pedestrian behaviors (PBQ). The questionnaire was received by 1,447 French participants, 610 of whom answered the entire questionnaire. Seventy-one percent of participants were women, and the mean age was 35 ± 14 years. Eye images give individuals the feeling they are being observed at 33%, feared at 5% and surprised at 26%, and thus seem to indicate mixed results about avoiding crossing at the red light. The expressions shown in the eyes are also an important factor: feelings of being observed increased by about 10-15% whilst feelings of being scared or inhibited increased by about 5% as the expression changed from neutral to friendly to angry. No link was found between the results of our questionnaire and those of the Pedestrian Behavior Questionnaire (PBQ). This study shows that the use of eye images could reduce illegal crossings by pedestrians, and is thus of key interest as a practical road safety tool. However, the effect is limited and how to increase this nudge effect needs further consideration.

## 1. Introduction

Whatever the size and complexity of the society we live in, social life involves respecting rules or norms in order to maintain peace and cohesion ( Coleman, 1994; Elster, 1989; Fehr and Fischbacher, 2004). Violating these social norms can unbalance the public good insofar that law-breakers will gain more benefits than their honest counterparts, or other individuals will be put at risk. In order to balance costs and benefits, punishment or police behaviors have evolved in humans societies ( Fehr and Gächter, 2000, 2002), and in other primate societies ( Boyd and Richerson, 1992; Flack *et al.*, 2006; Mendes *et al.*, 2018; Riedl *et al.*, 2012). Like other primate species, humans have developed emotional bases for prosocial behaviors allowing cooperation. These emotions are concerns, empathy and a sense of morality and of reputation ( De Waal, 2006; Jensen *et al.*, 2014; Keltner and Anderson, 2000; Penner *et al.*, 2005; Tomasello and Vaish, 2013) and they are defined as moral emotions by Haidt (2003). Being concerned or empathic enables humans to recognize when they are doing something wrong and correct their behavior in order to maintain a prosocial reputation and continue interacting cooperatively with their conspecifics ( Alexander, 1987; Bateson *et al.*, 2006; Burnham and Johnson, 2005).

Our behavior is therefore influenced by the presence of others, which scientists also call ‘the audience effect’ ( Filiz-Ozbay and Ozbay, 2014; Kurzban *et al.*, 2007; Zuberbühler, 2008). Feeling observed by real persons or an imagined audience, therefore has an impact on human behavior. Embarrassment, for instance, is defined as the ‘acute state of flustered, awkward, abashed chagrin that follows events that increase the threat of unwanted evaluations from real or imagined audiences’ ( Eller *et al.*, 2011; Miller, 1996). Being observed also tends to make individuals more compliant ( Dear *et al.*, 2019; Rodriguez Mosquera *et al.*, 2011). With this in mind, Bateson *et al.* (2006) conducted an experiment to test the effect of eye images on cooperative behavior. In the coffee area of a building at the University of Newcastle, an honesty box was used for people to make contributions to the coffee fund. The experiment consisted of placing pictures of eyes or flowers close to this box and assessing whether they led to differences in the contributions made. The authors found that in the presence of eye images, subjects paid on average 2.76 times more than when flowers images were displayed. Still comparing the effect of these flowers images as control to those of eye images, other studies have found similar prosocial effects during everyday events. For example, eye images had prosocial effects such as cooperation for the clearing of trays in a university cafeteria ( Ernest-Jones *et al.*, 2011) and for waste sorting at a bus stop ( Francey and Bergmüller, 2012). Similar results were found in experiments in more specific contexts such as blood donations: While eye images on flyers did not result in differences in pledge with a logo as control, more “real” donations were made by students who got the flyers with eyes image ( Sénémeaud *et al.*, 2017). Other than the activation of a “sense of being seen” ( Pfattheicher and Keller, 2015) or the desire to maintain a pro-social reputation ( Bateson *et al.*, 2006), it is important to note that humans possess neurons that respond to faces and eyes and activate such prosocial behaviors ( Emery, 2000; Haxby *et al.*, 2000). Bateson *et al.* (2013) suggest that eye images induce more pro-social behavior regardless of local norms, thus suggesting that the application of eye images could be a means to combat anti-social behavior by triggering a feeling of shame ( Nugier *et al.*, 2007).

The use of social control to produce more cooperative behaviors may positively influence road use and safety. The limitation of conflicts and accidents on road infrastructures is directly dependent upon the respecting of rules by the numerous pedestrians and drivers. However, more than 8000 pedestrians die in road accidents in Europe every year, 25% of whom die when using crosswalks ( Guéguen *et al.*, 2015, 2016). These lethal accidents are due to cars not stopping at signalized intersections but also to pedestrians crossing illegally at the red signal ( Sueur *et al.*, 2013). Past studies show that individuals do not cross illegally when other pedestrians are present ( Pelé *et al.*, 2017, 2019a, 2019b). Visual communication between two individuals can lead to a change in the receiver's behavior and this effect can be found in a road context. For example, eye contact is an important element. Some studies explored the effect of gaze and smile on the propensity of drivers to stop at signalized intersections and allow pedestrians to cross ( Guéguen *et al.*, 2015, 2016). A pedestrian waiting at the edge of an unmarked crosswalk has a greater likelihood to cross if s/he seeks to make visual contact with an approaching driver than if s/he is not looking towards the approaching car, with 67.7% of cars stopping versus 55.1%, respectively ( Guéguen *et al.*, 2015). If in addition to this visual contact the pedestrian smiles, 62.9% of drivers stopped compared to 50.1% if the pedestrian sought visual contact with a neutral face ( Guéguen *et al.*, 2016). These studies show that visual contact can modify the behavior and speed of drivers, and highlight that the facial expression of the pedestrian also has an impact ( Ren *et al.*, 2016). However, these studies are rare and more research is needed on how human facial expressions affect the probability that pedestrians will cross the road illegally. This research may have great potential in terms of applications in the field of road safety, especially regarding the regulation of pedestrian behaviors.

**Figure 1.**
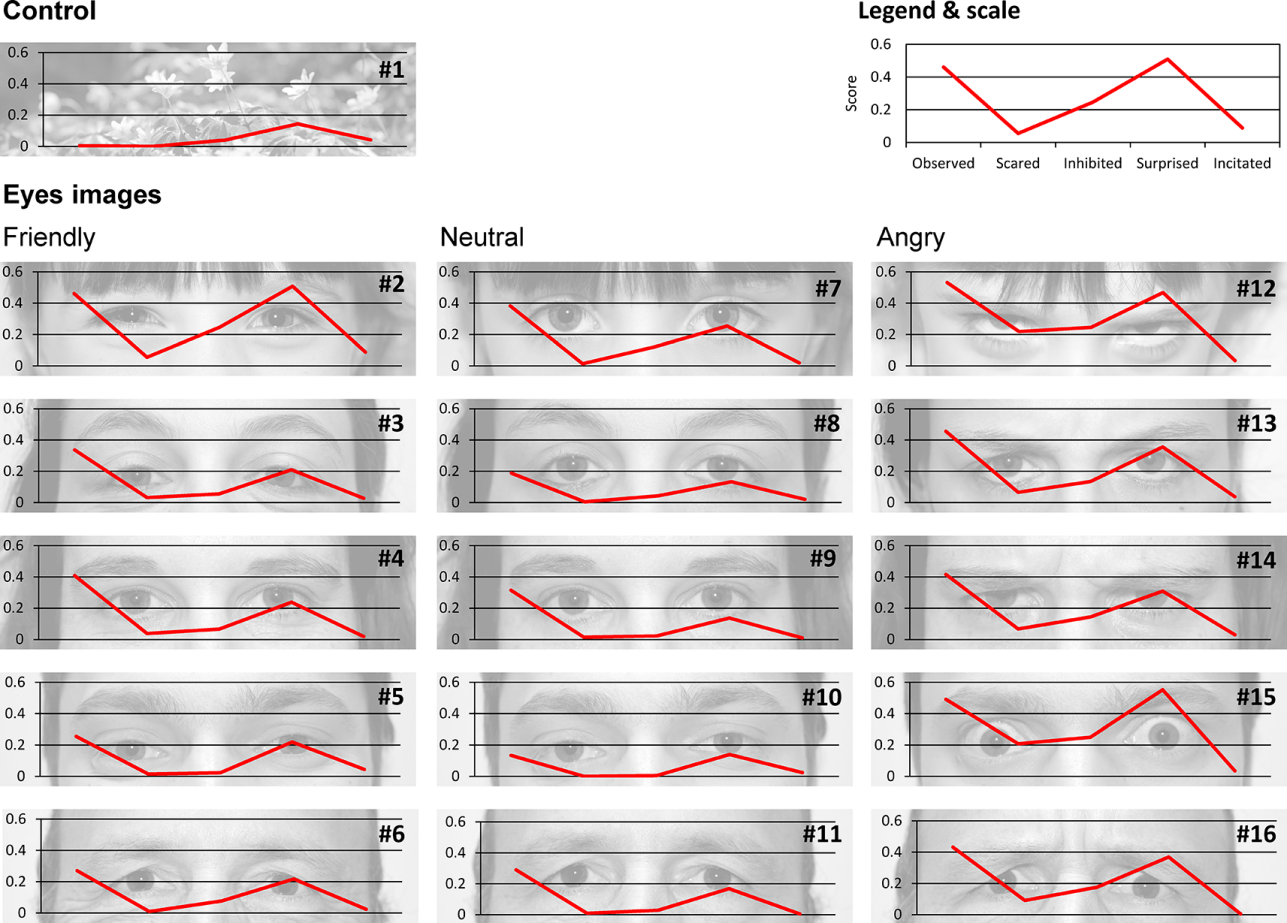
Images used to understand the effect of eye images on pedestrian behaviors. The flower image is a control. The effect of eye images was tested using three different expressions (friendly, neutral and angry respectively from left to right) in the eyes of five different persons. Five questions were asked about feelings for each eye image: Do you feel observed, scared, inhibited, surprised or encouraged? (See methods for details). The score for each feeling and each image is the average score for all participants. Image credits: Cédric Sueur.

This study aims to test the effect of eye images on the behaviors of pedestrians crossing at the red light. We collected different images of eyes from five different persons (two men, two women and one child) expressing different facial expressions (neutral, friendly and angry) and one image of flowers for use as a control ( Figure 1). These images were used in an online questionnaire that asked participants what they would feel if they saw these images before crossing a road. Would they feel observed, scared or surprised? Would the images discourage or encourage them to cross at the red light? These questions were chosen with caution in order to make the questionnaire valid and are based on previous studies ( Bateson *et al.*, 2013; Ernest-Jones *et al.*, 2011; Francey and Bergmüller, 2012; Saeed *et al.*, 2020). We compared their answers to these questions with sociodemographic variables (gender, age, geographical zone and city size) and also with a previous well-known questionnaire called ‘Pedestrian Behavior Questionnaire’ (hereafter referred to as PBQ, Appendix A: Deb *et al.*, 2017; Granié *et al.*, 2013), which tested the propensity of pedestrians to violate rules, make errors or lapses, or show positive or aggressive behaviors. The angry eye images are expected to have a stronger emotional impact on participants and thus prevent them from crossing illegally ( Bateson *et al.*, 2006). We also expect a gender effect and an age effect, with a stronger impact of eye images on women and younger individuals. Indeed, women might feel more observed or scared than men when looking at the eye images; studies have shown women to be more empathic and thus more receptive in different situations ( Flynn, 2000; Furnham *et al.*, 2003; Kellert & Berry, 1987; Miller *et al.*, 2009). In the context of road crossing, fewer illegal and risky behaviors are observed in women than in men ( Holland and Hill, 2007; Pelé *et al.*, 2017; Sueur *et al.*, 2013; Tom and Granié, 2011), with more positive behaviors and fewer rule violations or aggressive behaviors ( Deb *et al.*, 2017; Tom and Granié, 2011). Studies show that younger people are less respectful of rules ( Holland and Hill, 2007; Pelé *et al.*, 2017; Pfeffer and Hunter, 2013). We also expect regional differences in reactions to eye images, as interregional differences were observed in road accidents ( Eksler *et al.*, 2008; Lassarre and Thomas, 2005). Correlations are expected between the responses to the eye image questionnaire and the PBQ. Indeed, participants showing higher scores for violations or aggressive behaviors in the PBQ (i.e. the less prosocial individuals) should be less affected by eye images than participants who show more positive behaviors (i.e., the more empathic and cooperative individuals).

## 2. Methods

### 2.1 Questionnaire

The questionnaire was designed in three steps: 1. Eye images, 2. Pedestrian behavior questionnaire and 3. Sociodemographic questions *.*

*Eye images*: To test the effect of eye images, pictures were taken of five different individuals (one child, two women and two men) with three different facial expressions (friendly, neutral and angry). A picture of flowers was used as a control. Each picture was taken in black and white with the same contrast and brightness calibration (see Figure 1). For each of the 16 pictures (15 eyes and 1 control), participants were asked to answer five questions about their feelings when looking at the picture:

1. Observation: Do you feel observed when looking at this picture? Answer: yes/maybe/no
2. Fear: Are you scared when looking at this picture? Answer: yes/maybe/no
3. Inhibition: Would looking at this picture prevent you from crossing at the red light? Answer: yes/maybe/no
4. Surprise: Would this picture make you feel surprised if you saw it before crossing the road? Answer: yes/maybe/no
5. Incitation: Would seeing this picture encourage you to cross at the red pedestrian signal? Answer: yes/maybe/no

These five questions were chosen with caution. In order to check the validity of the questionnaire, we chose the four first questions to evaluate the negative effects of the eyes images and the fifth question to evaluate a positive effect. This means that we expected to have the scores of the fourth first questions to be positively correlated between them but negatively correlated with the fifth question. The questionnaire was validated with results ( Section 3.1.) following our predictions.

As five questions for 16 pictures results in a very long questionnaire of 80 questions, 4 images (with for each image the five questions) were randomly selected for the participants to answer. We checked for a homogeneous distribution of questions to participants by making packages of 20 questions (for 4 images).

*Sociodemographic questions:* Participants were asked to indicate their gender (female or male), age (as numeric) and French postal code. The French postal code provides the city population size (DataNova Opensource, 2014 census) and the Defense and Security Zones (French Homeland Ministrysee https://doi.org/10.5281/zenodo.5745446.

*Pedestrian behavior questionnaire (PBQ):* Participants completed a questionnaire of 20 questions about pedestrian behaviors by Deb *et al.* (2017). We used a pedestrian behavior questionnaire (PBQ) with 20 questions, developed by ( Deb *et al.*, 2017) and taken from ( Tom and Granié, 2011). The order of questions was randomly attributed. In parenthesis, the average±stdv of the score (from 1 – never - to 6 – always -) attributed by participants to each question (N=611).

**Violations (V)**

V1 I cross the street even though the pedestrian light is red. (3.73 ± 1.35)

V2 I cross diagonally to save time. (3.45 ± 1.42)

V3 I cross outside the pedestrian crossing even if there is one (crosswalk) less than 50 m away. (3.51 ± 1.49)

V4 I take passageways forbidden to pedestrians to save time. (2.35 ± 1.36)

**Errors (E)**

E1 I cross between vehicles stopped on the roadway in traffic jams. (3.34 ± 1.43)

E2 I cross even if vehicles are coming because I think they will stop for me. (2.36 ± 1.19)

E3 I walk on cycling paths when I could walk on the sidewalk. (1.91 ± 0.96)

E4 I run across the street without looking because I am in a hurry. (1.27 ± 0.63)

**Lapses (L)**

L1 I realize that I have crossed several streets and intersections without paying attention to traffic. (1.52 ± 0.82)

L2 I forget to look before crossing because I am thinking about something else. (1.65 ± 0.78)

L3 I cross without looking because I am talking with someone. (1.65 ± 0.85)

L4 I forget to look before crossing because I want to join someone on the sidewalk on the other side. (1.45 ± 0.70)

**Aggressive Behaviors (A)**

A1 I get angry with another road user (pedestrian, driver, cyclist, etc.), and I yell at him. (1.94 ± 1.17)

A2 I cross very slowly to annoy a driver. (1.45 ± 0.87)

A3 I get angry with another road user (pedestrian, driver, cyclist, etc.), and I make a hand gesture. (2.08 ± 1.20)

A4 I have gotten angry with a driver and hit their vehicle. (1.25 ± 0.70)

**Positive Behaviors (P, Reverse-scaled items)**

P1 I thank a driver who stops to let me cross. (5.55 ± 0.82)

P2 When I am accompanied by other pedestrians, I walk in single file on narrow sidewalks so as not to bother the pedestrians I meet. 4.05 ± 1.46)

P3 I walk on the right-hand side of the sidewalk so as not to bother the pedestrians I meet. (4.04 ± 1.48)

P4 I let a car go by, even if I have the right of way, if there is no other vehicle behind it. (3.60 ± 1.49)

Questions were presented in random order. PBQ was the first complete questionnaire to study a broad range of aspects of pedestrian behavior on the road for all age groups. This questionnaire was originally developed by Tom and Granié (2011) with 47 questions. We chose the short version, which is considered as reliable as the long version according to Deb *et al.* (2017) and Tom and Granié (2011), in order to avoid demotivating participants. The 20 questions are categorized into five items as followed:

1. Transgression: deliberate deviation from social rules without intention to cause injury or damage, Reason *et al.*, 1990;
2. Error: deficiency in knowledge of traffic rules and/or in the inferential processes involved in making a decision, Rasmussen, 1980; Reason *et al.*, 1990;
3. Lapse: unintentional deviation from practices related to a lack of concentration on the task; forgetfulness, Reason *et al.*, 1990;
4. Aggressive Behavior: tendency to misinterpret other road users’ behavior, resulting in the intention to annoy or endanger, Baxter *et al.*, 1990; Lawton *et al.*, 1997;
5. Positive Behavior: behavior that seeks to avoid violation or error and/or seeks to ensure traffic rule compliance, Özkan and Lajunen, 2005.

The participants were required to answer the questions using a 6-point Likert scale (1-very infrequently or never, 2-quite infrequently, 3-infrequently, 4-frequently, 5-quite frequently, 6-very often or always).

### 2.2 Survey administration and participants

The survey was created using LimeSurvey ( Engard, 2009; Jayasundara *et al.*, 2010; LimeSurvey Project Team, 2012) and administered online to the French population through mails and social media (Facebook and Twitter).

The questionnaire was received by 1,447 participants, 610 of whom answered the entire questionnaire (all three steps). The resulting dataset was used in our analyses (N=610). The mean time to answer the entire questionnaire was seven minutes. 71% of participants were women, and the mean age was 35 ± 14 years. The geographic repartition of the population is as follows: Hauts-de-France (N=17), Ile-de-France (N=100), Ouest (N=92), Est (N=219), Sud-Ouest (N=18), Sud-Est (N=75), Sud (N=89). These factors were included in statistical analyses to avoid selection biases selection.

### 2.3 Research ethics

All data were anonymous, and participants were given sequential numerical identities corresponding to the moment they answered the questionnaire. Participants could obtain information about the study and its results by contacting the authors via an email address provided at the end of the questionnaire. We followed the ethical guidelines of our institution (CNRS-IPHC, Strasbourg, France). This study received ethical approval from the road security direction of the French Homeland Ministry (RefCNRS190529).

Formal written agreement or parental consent was obtained from the five people photographed for the eye images. They were aware of, and agreed to, the intended use of the photographs in the questionnaire and in the publication.

### 2.4 Statistical analyses

We first calculated the mean score for each question and each image. This score ranges from 0 to 1, where 0 indicates 100% of participants answering “No” to the question and 1 corresponds to 100% answering “Yes”. The scores concerning all three answers [Yes, maybe, No] and the scores concerning only two answers [Yes, No] are correlated at 95% (linear regression, P<0.0001, R ^2^ = 0.95, N = 80). For this reason and in order to simplify statistical analyses, we only used the [Yes, No] answers.

A Pearson correlation test was used to check the correlation between each feeling. We then performed a principal component analysis (PCA), followed by Hierarchical Clustering on Principal Components (HCPC) in order to assess which images resulted in higher scores. Following these analyses, the incitation question was excluded from the next statistical tests (see results) and a mean score was calculated for each participant, combining all four remaining questions.

We assessed whether this score is influenced by the age, gender, geographical zone of their city or the city population size (categorized according to quartiles). A general linear model (glm) with a normal law was used with the R package “MultComp” for multiple comparisons ( Bretz *et al.*, 2016). A separate GLM tested the interactions between age/sex factors and the expression of eye images (i.e., neutral, angry, friendly). The conditions of application (normality and homoscedasticity of residuals) were graphically verified.

The 20 PBQ questions with values from 1 to 6 were analyzed using a PCA with a varimax rotation (Package R Psych; Revelle, 2011; Revelle & Revelle, 2015), following the procedure explained in Granié *et al.* (2011, 2013). In order to fit with the PCA axes of these studies, we set a maximum number of four loadings (as a preliminary analysis of five PCA dimensions shows a division of the positive behaviors in dimensions 4 and 5, we combined both dimensions as described by Granié *et al.*, 2011, 2013). The coordinates of participants in each dimension (five with loadings higher than 1.00) were then compared with the eye images mean score using a Pearson correlation test. GLM analysis was also carried out to test coordinates with gender, age and city data of participants using. The four dimensions were scaled and normalized using the scale function in R. For the “zone” variable, an Anova followed by a Tukey posthoc test was performed on the GLM residuals.

All tests were carried out with R 3.6 ( R Development Core Team, 2009). The significance level was set at 0.05. Results are indicated with mean ± stdv.

## 3. Results

### 3.1 What do participants feel when seeing the eye images?

Whatever the eye image, the mean score for the ‘Observation’ question is 0.33 ± 0.14 (meaning that 33% of participants answered ‘Yes’ to this question and therefore feel observed). The mean score for the ‘Fear’ question is 0.05 ± 0.07. The mean score for the ‘Inhibition’ question is 0.10 ± 0.08. The mean score for the ‘Surprise’ question is 0.26 ± 0.12. Finally, the mean score for the ‘Incitation’ question is 0.02 ± 0.01. Scores for each image and each question are provided in Table 1 and are shown in Figure 1.

**Table 1.** Mean score for each eye image (except the flower control image) and each feeling-related question. Images are ranked according to their values in dimension 1 of the PCA, without the incitation variable. Colors indicate the clusters assessed by the HCPC, from the least intense (green) to the most intense (red) feeling.

Category	Facial expression	Observation	Fear	Inhibition	Surprise	Incitation	PCA Dim1 Coord
Flower	-	0.00	0.00	0.04	0.14	0.04	-2.45
Man	Neutral	0.13	0.00	0.00	0.14	0.02	-2.25
Woman	Neutral	0.19	0.00	0.04	0.13	0.02	-1.80
Woman	Neutral	0.31	0.01	0.02	0.14	0.01	-1.41
Man	Neutral	0.29	0.01	0.03	0.17	0.00	-1.37
Man	Friendly	0.26	0.02	0.02	0.22	0.04	-1.29
Man	Friendly	0.27	0.01	0.08	0.22	0.02	-0.95
Woman	Friendly	0.34	0.03	0.06	0.21	0.03	-0.72
Woman	Friendly	0.41	0.04	0.07	0.24	0.02	-0.25
Child	Neutral	0.38	0.01	0.12	0.25	0.02	-0.10
Woman	Angry	0.42	0.07	0.14	0.31	0.03	0.75
Woman	Angry	0.46	0.07	0.13	0.36	0.04	1.01
Man	Angry	0.43	0.09	0.18	0.37	0.00	1.41
Child	Friendly	0.46	0.06	0.25	0.51	0.09	2.24
Child	Angry	0.53	0.22	0.25	0.47	0.03	3.52
Man	Angry	0.49	0.21	0.25	0.55	0.04	3.66

**Figure 2.**
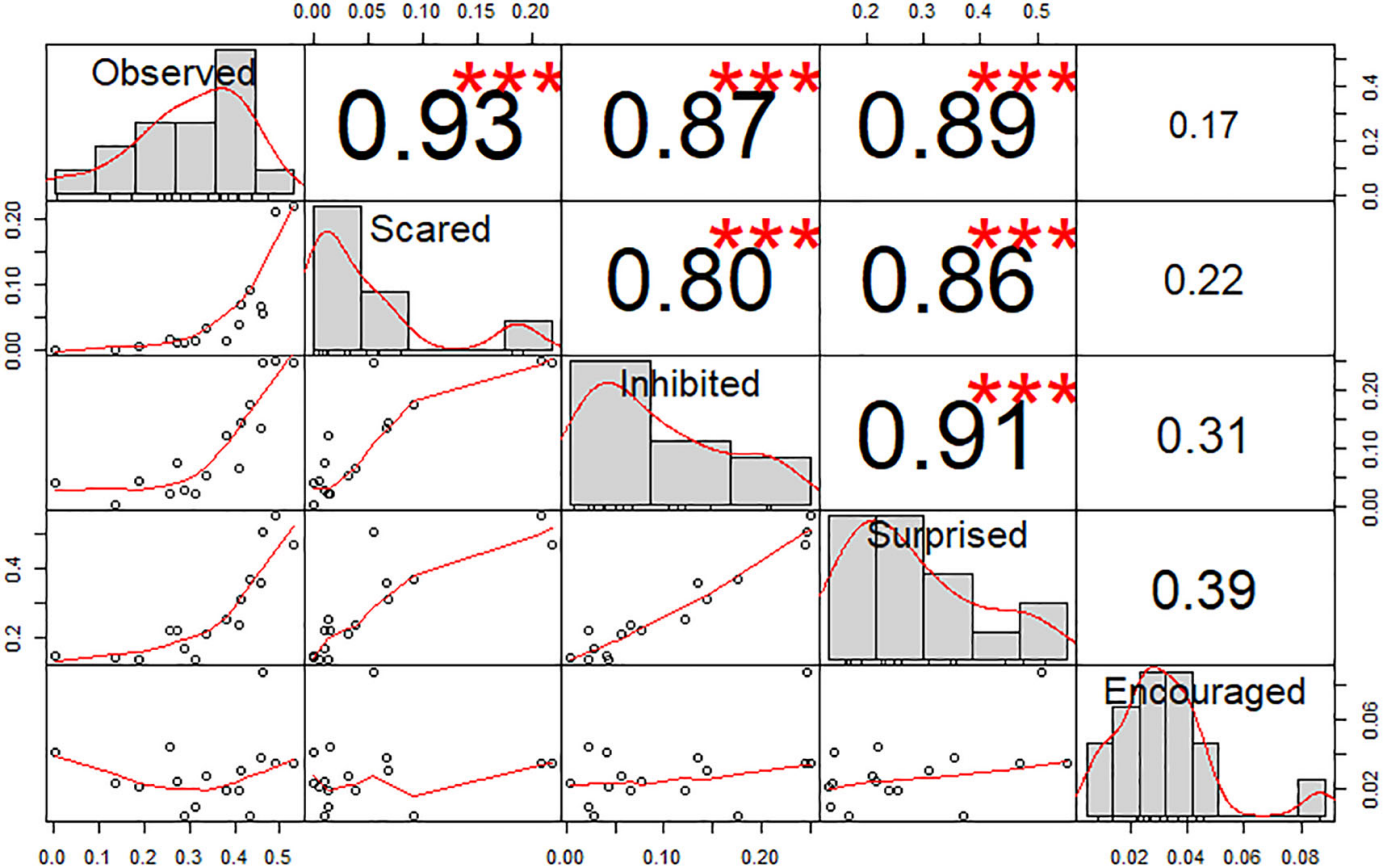
Chart of correlations between the feelings scores. Values indicate the Pearson correlation. Stars indicate statistical significance (Absence = p > 0.05; *** = p < 0.001).

Pearson’s correlation tests between scores ( Figure 2) showed a high correlation between all feelings (r > 0.8, p < 0.001) excluding incitation (r < 0.39, p > 0.05). Indeed, a PCA showed that dimension 1 was composed of four feelings (Observation, Fear, Inhibition and Surprise, r > 0.85see https://doi.org/10.5281/zenodo.5745446 for details) and explained 72.73% of variance in the scores, whilst dimension 2, which was solely composed of the Incitation feeling (r = 0.89), explained 19.28% of variance. These results validate our questionnaire with participants answering coherently following our predictions. A HCPC following this PCA produced five clusters, without including picture 2 (Child’s friendly eyes). When the incitation question was removed from analyses, dimension 1 (r > 0.88) explained 87.06% of score variance whilst dimension 2 (r < 0.45) explained only 7.28%. The resulting HCPC produced four clusters, with picture 2 regrouped with another cluster. In view of these results and the aims of our study, we decided to discard the incitation question for the following analyses. The images were then grouped into four clusters. The first cluster includes the flower, and a man and a woman with neutral expressions. It has a lower influence on the scores (see green elements in Table 1; participants do not feel surprised, scared, observed or inhibited). The fourth and last cluster (see red elements in Table 1) is composed of the child and a man with an angry expression. This cluster shows the highest scores (i.e. participants felt observed, scared, surprised and inhibited). Gender is therefore equally distributed in these four clusters, but the picture of a child’s eyes has a strong effect on feelings (ranked 10, 14 and 15 on the 16 images, Table 1). Moreover, whilst the first cluster includes only ‘neutral’ images and the second cluster includes ‘friendly’ and ‘neutral’ images, all the angry images are found in the third and fourth clusters, meaning that these expressions lead to stronger feelings.

### 3.2 How did the influence of eye images on the feelings of participants vary according to their sociodemographic factors?

**Figure 3.**
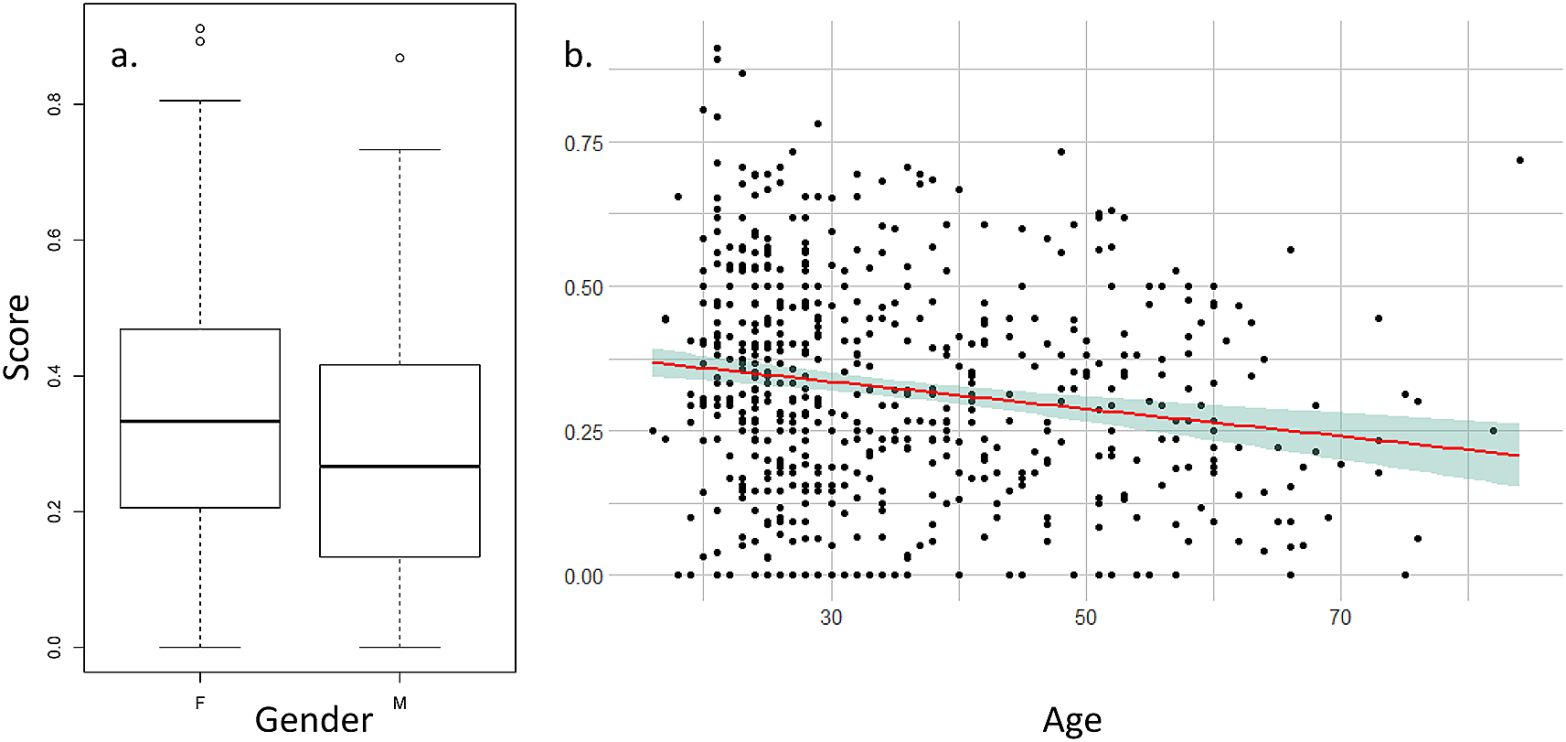
Influence of gender (a.) and age (b.) on the mean score of the feelings of participants on seeing the eye images.

The mean score for each participant was influenced by gender and age ( Figure 3) but not by the geographical zone or the city population size (see Table 2 for statistical values). Men have a lower score than women, meaning that they feel less scared or observed by eyes. Age negatively influences the score, meaning that older participants have a lower score and feel less observed or scared than youngers. When we tested the effect on the mean score of the interactions of age and sex with the eye expressions (i.e., neutral, angry or friendly), we did not found any interaction between age and eye expression (|t-value| < 1.086, p > 0.277) or between sex and eye expression (|t-value|< 1.449, p > 0.147).

**Table 2.** Statistical values for the general linear model with the mean score of the eye images questionnaire. HDF for Hauts-de-France, IDF for Ile-de-France.

	Estimate	Std. Error	z value	Pr(>|z|)
(Intercept)	0.4071361	0.0306555	13.281	<0.001
Gender [Male]	-0.050761	0.0164812	-3.08	0.0198
Age	-0.0022274	0.0005603	-3.975	<0.001
ZoneHDF	0.0089592	0.0461934	0.194	1
ZoneIDF	0.0398962	0.0221828	1.799	0.4841
ZoneOuest	0.0538668	0.023243	2.318	0.172
ZoneSud	0.0259914	0.02306	1.127	0.9272
ZoneSudEst	-0.0156868	0.0246333	-0.637	0.9986
ZoneSudOuest	-0.0018212	0.0449061	-0.041	1
Population size	-0.0030641	0.0066881	-0.458	0.9999

### 3.3 Answers of participants to the pedestrian behavior questionnaire

The 20 PBQ questions were analyzed in the same way as those in referenced studies ( Deb *et al.*, 2017; Granié *et al.*, 2013; Tom and Granié, 2011). Results are in accordance with the precited studies (see https://doi.org/10.5281/zenodo.5745446 for details). The results explain 49.7% of total variance. The Kaiser-Meyer-Olkin measure of sampling adequacy was satisfactory (0.80) and the Bartlett’s test of sphericity was significant (p < 0.0001). The first dimension explains 15.7% of variance and corresponds to “lapses” (following a loading above 0.4). The second dimension explained 14.7% of variance and corresponds to “transgressions”, meaning violations and errors. The third dimension explains 11.2% of variance and corresponds to “aggressive behaviors”. Finally, the fourth dimension explains 8.2% of variance and corresponds to “positive behaviors”.

### 3.4 How did the PBS axes influence participants according to their sociodemographic factors?

All statistical results are indicated in Table 3. Posthoc Tukey multiple comparisons are detailed in https://doi.org/10.5281/zenodo.5745446. The “lapses” dimension is only influenced by the city population size, with a larger city population size leading to higher occurrence of unintentional deviation from rules. The “transgressions” dimension (violations and errors) is influenced by age, with younger people making more transgressions, and also by geographical zones, with a higher transgressions score for the “Sud” (South) Zone than the “Est” (East) area (however, see the Tukey test for further details). The “aggressive behaviors” axis is influenced only by the zone but the posthoc test revealed no differences. Finally, the positive behaviors dimension is influenced by gender, with women showing more positive behavior than men do.

**Table 3.** Statistical values of the general linear models concerning the PBQ dimensions. HDF for Hauts-de-France, IDF for Ile-de-France. QuartilePop indicates size of towns populations as quartiles.

	Estimate	Std. Error	t value	Pr(>|t|)
**Lapses dim.**				
(Intercept)	0.9604247	0.027685	34.691	<2e-16
Gender [Male]	-0.0289539	0.0177072	-1.635	0.1025
Age	-0.0002319	0.0003504	-0.662	0.5083
ZoneHDF	-0.0567546	0.0495352	-1.146	0.2524
ZoneIDF	-0.0104238	0.0238461	-0.437	0.6622
ZoneOuest	0.0060215	0.0249557	0.241	0.8094
ZoneSud	0.0128254	0.0247557	0.518	0.6046
ZoneSudEst	-0.0014063	0.0264376	-0.053	0.9576
ZoneSudOuest	0.0219626	0.0481632	0.456	0.6486
**QuartilePop**	**0.0142764**	**0.0071656**	**1.992**	**0.0468**
**Trangressions dim.**				
(Intercept)	0.9636292	0.0273093	35.286	<2e-16
Gender [Male]	0.0299833	0.0174669	1.717	0.08657
**Age**	**-0.0008977**	**0.0003456**	**-2.597**	**0.00962**
ZoneHDF	0.0116038	0.048863	0.237	0.81237
ZoneIDF	-0.0023133	0.0235225	-0.098	0.92169
ZoneOuest	-0.0323359	0.0246171	-1.314	0.1895
**ZoneSud**	**0.0659362**	**0.0244198**	**2.7**	**0.00713**
ZoneSudEst	0.0156855	0.0260788	0.601	0.54776
ZoneSudouest	-0.0083361	0.0475096	-0.175	0.86078
QuartilePop	0.0131719	0.0070683	1.864	0.06288
**Aggressive behav. dim.**				
(Intercept)	9.47E-01	2.77E-02	34.149	<2e-16
Gender [Male]	2.26E-02	1.77E-02	1.273	0.2037
Age	9.01E-05	3.51E-04	0.257	0.7976
ZoneHDF	3.28E-02	4.96E-02	0.661	0.5087
ZoneIDF	1.32E-02	2.39E-02	0.552	0.581
ZoneOuest	1.88E-02	2.50E-02	0.752	0.4521
**ZoneSud**	**5.82E-02**	**2.48E-02**	**2.345**	**0.0194**
ZoneSudEst	2.34E-02	2.65E-02	0.881	0.3785
ZoneSudOuest	3.34E-02	4.83E-02	0.693	0.4888
QuartilePop	1.69E-03	7.18E-03	0.235	0.814
**Positive behav. dim.**				
(Intercept)	1.00474	0.0276472	36.341	<2e-16
**Gender [Male]**	**-0.0399417**	**0.017683**	**-2.259**	**0.0243**
Age	-0.0005901	0.0003499	-1.686	0.0922
ZoneHDF	0.0104337	0.0494676	0.211	0.833
ZoneIDF	0.015133	0.0238136	0.635	0.5254
ZoneOuest	0.0030133	0.0249217	0.121	0.9038
ZoneSud	0.0129851	0.0247219	0.525	0.5996
ZoneSudEst	-0.0044673	0.0264015	-0.169	0.8657
ZoneSudOuest	0.057924	0.0480975	1.204	0.2289
QuartilePop	0.0006763	0.0071558	0.095	0.9247

### 3.5 Link between feelings on seeing the eye images and PBQ axes

**Figure 4.**
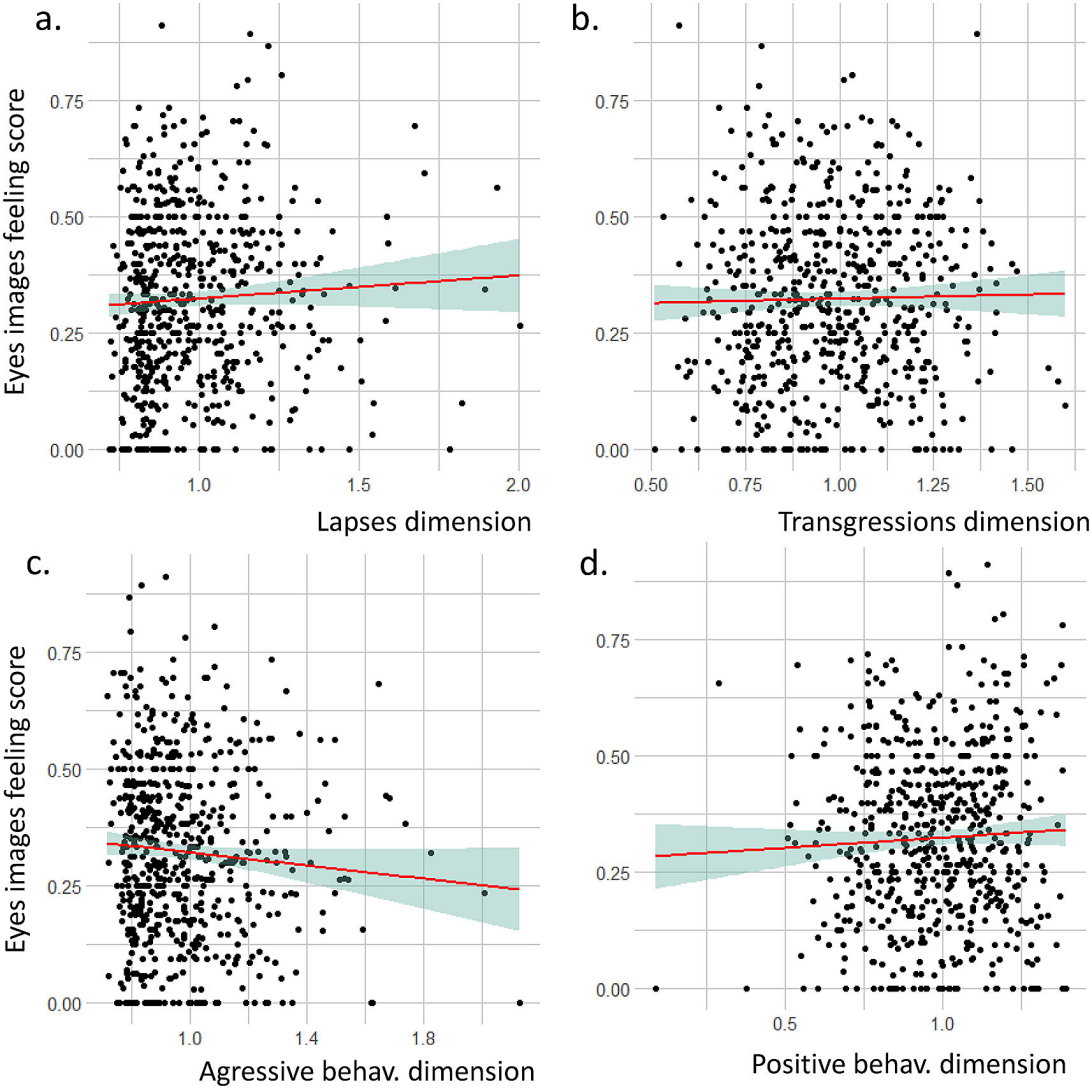
Relation between the eye image feeling score and the four dimensions of the pedestrian behavior questionnaire (PBQ).

We did not identify any statistical correlation between the eye image questionnaire score and the four PBQ dimensions ( Figure 4). Lapses dimension: r= 0.05, t = 1.2843, df = 608, p-value = 0.1995; Transgressions dimension: cor = 0.02, t = 0.47498, df = 608, p-value = 0.635, Aggressive behaviors dimension: r = -0.07, t = -1.8156, df = 608, p-value = 0.06993; Positive behaviors dimension: r = 0.05, t = 1.129, df = 608, p-value = 0.2593.

## 4. Discussion

This study tested the potential impact of different eye images – friendly, neutral, and angry –and one image of flowers as a control on pedestrians crossing at the red light. In their responses to an online questionnaire about their feelings on seeing these images, participants revealed that they initially felt observed (about 33%), then surprised (26%), then inhibited to cross at the red signal (10%). Finally, few were scared (5%) or felt encouraged to cross at the red signal (2%). Eye images encourage cooperative behavior because unlike pictures of flowers, they make participants feel like they are being watched ( Pfattheicher and Keller, 2015). Our results are in line with this explanation, as flowers obtained a null score for being observed or scared. Moreover, the expression conveyed by the eyes also affects participants, as feelings of being observed increased by about 10-15% whilst feelings of being scared or reluctant to cross the road increased by about 5% as the expression changed from neutral to friendly to angry. This study confirms that humans react to faces but rates of negative reactions to the eyes are low, indicated a mixed effect. Indeed, we expected more participants to answer that they feel observed, afraid or surprised by the eyes images. Previous studies have shown that this reaction can play a role in maintaining the cooperative behaviors that are essential to life in societies ( Alexander, 1987; Bateson *et al.*, 2006; Burnham and Johnson, 2005) and it is interesting to put in light our results with these studies.

As predicted by our hypothesis, we found that the gender and age of participants affected their feelings when looking at the eye images. Women were more affected by the images than men were, and younger participants also reacted more than older individuals. Few studies have analyzed the effect of these two variables on the reaction to eye images, maybe because of anonymity in questionnaires or the low number of participants. Two studies report that gender did not influence reactions to eye images ( Rodriguez Mosquera *et al.*, 2011; Sparks and Barclay, 2013). However, it is not surprising to find that women felt more observed or scared than men when looking at the eye images, as studies showed women to be more empathic and therefore more receptive in different situations ( Flynn, 2000; Furnham *et al.*, 2003; Kellert & Berry, 1987; Miller *et al.*, 2009). In the context of road crossing, women show also fewer illegal andrisky behaviors than men ( Holland and Hill, 2007; Pelé *et al.*, 2017; Sueur *et al.*, 2013; Tom and Granié, 2011). Women also show more positive behaviors, as shown in our study, and fewer violations or aggressive behaviors than men do ( Deb *et al.*, 2017; Tom and Granié, 2011).

Although we expected the effect of eye images on feelings to increase with age, the contrary was observed. To our knowledge, this is the first study on the effect of eye images to date that has reported such a result. However, studies of pedestrian behaviors showed younger people to be less respectful of rules ( Holland and Hill, 2007; Pelé *et al.*, 2017; Pfeffer and Hunter, 2013). This is confirmed in our study, which shows more violations by younger participants than by their older counterparts. Elder pedestrians can also display some illegal behaviors but these are generally due to cognitive or physical disorders ( Laurin *et al.*, 2001; Yaffe *et al.*, 2001). Contrary to past studies using PBQ ( Deb *et al.*, 2017; Granié *et al.*, 2013), we did not find an age effect on each pedestrian behavior axis. Our results show an effect of the city population size on the lapses dimension, meaning that citizens living in big cities show more unintentional violations and/or are more distracted than the inhabitants of small cities, probably because of the density of the population or visual distractions such as shops, signs or public transport. Participants from the South of France seem to display more violations and aggressive behaviors than those from the rest of France.

No link was found between the results of our questionnaire and those of the Pedestrian Behavior Questionnaire (PBQ). We expected participants showing high scores for violations and aggressive behaviors in the PBQ (or low scores for positive behaviors) to feel less observed or scared by the eye images. However, no correlation was found. This can be explained by a number of reasons. A participant may feel concerned by the eye image but will behave aggressively towards a driver because as a pedestrian, s/he considers that the driver is wrong (and thus seeks to communicate this anger). Alternatively, a participant may not feel concerned by the eyes image because irrespective of who is watching him/her, s/he will always behave well and consider the behavior of the driver - if the latter also behaves well - as reciprocal ( Burnham and Johnson, 2005; Fehr and Fischbacher, 2004). This absence of correlation may be due to differences in the empathic profiles of the population, ranging from people who are naturally cooperative and/or react to eye images in order to ensure they are seen in a positive light, to those who are not cooperative at all and are more likely to react to punishment ( Boyd and Richerson, 1992; Fehr and Gachter, 2000). Although we expected people who followed the rules to be non-violent, the two traits may not be correlated. In other words, a person may react to eye images and be cooperative or follow the rules but be aggressive towards people who do not do likewise. Conversely, another person may be indifferent to others, and will therefore not react to eye images or behave aggressively towards people who do not respect the rules. A second explanation is that the questionnaire is based on a virtual situation that did not affect participants’ feelings in the same way as the real situation, thus decreasing the potential correlation between our variables ( Francey and Bergmüller, 2012). Past studies have indeed shown a great variability of participants responding to eye images according to the experimental setup that is used (see for instance Fehr & Schneider, 2010; Northover *et al.*, 2017 for negative results). Anonymity also has a negative impact on the effect of eye images ( Lamba and Mace, 2010), and this could have an impact in our study.

## 5. Conclusion

A better understanding of human cooperative behavior in real life is of key interest for social management, from both theoretical and practical perspectives. The present study shows that the use of eye images could help to reduce illegal crossings by pedestrians. However, this effect is limited as not a majority of participants answered that they felt observed, afraid and so on. Drivers react to the smiles and gaze of pedestrians by permitting them to cross a road ( Guéguen *et al.*, 2015, 2016). Pedestrians behaving in this way could also be more cooperative. The mechanisms involved in maintaining a good reputation can also produce investments to serve the common good ( Bshary and Bshary, 2010; Francey and Bergmüller, 2012). Our findings are of practical interest for those designing honesty-based systems, or wishing to maximize contributions to public commodities and services. In a meta-analysis of 15 experiments from 13 research papers ( Dear *et al.*, 2019), found a 35% reduction in the risk of antisocial behavior when eye images are present. In contrast, systematic reviews have suggested that CCTV cameras reduce crime by only 16%. Settling such eyes images nudges on pedestrian signals could have an effect, even a small one, but this could be enough to decrease significantly accidents, particularly considering the group effect of crossings ( Pelé *et al.*, 2017). However, how such effect persist in time as pedestrians could get habituated to the eyes as reprimand is absent. However, our study is based on a questionnaire and this nudge needs to be tested in real situations. We encourage authorities to adopt the use of eye image systems in crossing signals in order to decrease the number of illegal crossings and increase pedestrian safety. Field research as well as more ecologically valid situations must be added to laboratory-based studies to show the real effect of these eye images on human cooperative behaviors.

## Data availability

Zenodo. Dataset for Eye image effect in the context of pedestrian safety: a French questionnaire study. DOI: https://doi.org/10.5281/zenodo.5745446
