# Supplementary material for: Eye image effect in the context of pedestrian safety: a French questionnaire study
Source: F1000Res. 2025 Aug 22;11:218. Originally published 2022 Feb 23. [Version 3] doi: 10.12688/f1000research.76062.3 (PMC10562793; doi:10.12688/f1000research.76062.3)
Supplement: Peer review history

# Reviewer report: Reviewer response for version 3

**DOI:** 10.5256/f1000research.186964.r408797

**Published:** 2025-09-22

**Authors:** Muhammad Faizan ul Haq

**Affiliations:** 1 Nagoya University, Nagoya, Japan

The manuscript provides valuable insights into the state of the art of pedestrian behavior research, particularly regarding the impact of eye images on pedestrian behavior. It is generally well prepared and contributes meaningfully to the literature. However, I believe the paper could be further improved before publication. My comments are as follows:

 Major and Minor Comments

1. **Section 1.3:**
  The authors used a flower picture as a control case. Could the rationale for this choice be explained more clearly? A blank or neutral image might serve as a more straightforward control. Please clarify why the flower image was considered appropriate.
2. **Section 2.1:**
  The following sentence would benefit from restructuring for clarity:
*“We expected the scores of the first four questions to be positively correlated with each other, but negatively correlated with the fifth question.”*
  This revision makes the intended meaning clearer and grammatically precise.
3. **Section 2.2:**
  The reported standard deviation of the population size is 237,535 ± 252,149 (min = 46; max = 2,220,445). A minimum city population of 46 seems highly unrealistic for such a study. The authors should revisit this value, verify data quality, and explain whether it is a reporting error or an outlier that requires justification.
4. **Table 1:**
  The meaning of the different color segregations in the table is not clearly explained. Additionally, some categories appear repetitive (e.g., “Man Neutral” and “Man Friendly”), which creates confusion. More detailed illustration, clearer labeling, or supplementary explanation in the text would help improve the interpretability of this table.
5. **Figure 3:**
  Since 71% of the sample consisted of female participants, gender distribution may significantly influence the results. It would strengthen the analysis to examine age effects separately for men and women, rather than analyzing the entire sample together. This would help address the imbalance and provide more nuanced insights.

Is the work clearly and accurately presented and does it cite the current literature?

Yes

If applicable, is the statistical analysis and its interpretation appropriate?

Yes

Are all the source data underlying the results available to ensure full reproducibility?

Partly

Is the study design appropriate and is the work technically sound?

Yes

Are the conclusions drawn adequately supported by the results?

Yes

Are sufficient details of methods and analysis provided to allow replication by others?

Yes

Reviewer Expertise:

Road user safety, transportation planning, behaviour modelling

I confirm that I have read this submission and believe that I have an appropriate level of expertise to confirm that it is of an acceptable scientific standard, however I have significant reservations, as outlined above.

# Reviewer report: Reviewer response for version 2

**DOI:** 10.5256/f1000research.152142.r209190

**Published:** 2023-10-09

**Authors:** Yunchang Zhang

**Affiliations:** 1 Desay SV, Singapore, Singapore

I think the authors have already addressed my comments

Is the work clearly and accurately presented and does it cite the current literature?

Yes

If applicable, is the statistical analysis and its interpretation appropriate?

Yes

Are all the source data underlying the results available to ensure full reproducibility?

Partly

Is the study design appropriate and is the work technically sound?

Yes

Are the conclusions drawn adequately supported by the results?

Partly

Are sufficient details of methods and analysis provided to allow replication by others?

Yes

Reviewer Expertise:

Social Behavior, human interactions.

I confirm that I have read this submission and believe that I have an appropriate level of expertise to confirm that it is of an acceptable scientific standard.

# Reviewer report: Reviewer response for version 2

**DOI:** 10.5256/f1000research.152142.r209191

**Published:** 2023-10-09

**Authors:** Rahul Raoniar

**Affiliations:** 1 Indian Institute of Technology Guwahati, Guwahati, Assam, India

The authors have effectively integrated the recommended changes and have substantiated them with sound justifications backed by evidence.

 I would suggest one further addition to the discussion and study limitations section.

 While acknowledging that road safety encompasses various dimensions, including psychological and social interventions, whose efficacy may vary, it is crucial to underscore the significance of infrastructural design modifications. Prioritizing such changes can greatly enhance pedestrian safety by eliminating the need for risky maneuvers when crossing roads.

Is the work clearly and accurately presented and does it cite the current literature?

Yes

If applicable, is the statistical analysis and its interpretation appropriate?

Yes

Are all the source data underlying the results available to ensure full reproducibility?

Yes

Is the study design appropriate and is the work technically sound?

Yes

Are the conclusions drawn adequately supported by the results?

Yes

Are sufficient details of methods and analysis provided to allow replication by others?

Partly

Reviewer Expertise:

Walkable and Cyclable Cities, Road Safety, Injury Prevention, Transport Psychology, Transportation Accessibility, and Quantitative Social Research.

I confirm that I have read this submission and believe that I have an appropriate level of expertise to confirm that it is of an acceptable scientific standard.

# Reviewer report: Reviewer response for version 1

**DOI:** 10.5256/f1000research.80016.r172758

**Published:** 2023-05-25

**Authors:** Rahul Raoniar

**Affiliations:** 1 Indian Institute of Technology Guwahati, Guwahati, Assam, India

The study focuses on exploring the effect of eye image on pedestrian road crossing behavior (signal violation). The overall work is good; still, the authors need to address the following comments to strengthen the manuscript further:

**Abstract**

 None

**Introduction**

 Human eye contact, facial expressions (smile), and hand gestures could lead to changes in driver behavior (page 3 of the pdf) and also helps pedestrian to cross busy roads.

- These might not be the only factors that determine the signal violation behavior. There are numerous factors like non-social (traffic volume, composition, infrastructure) and social (presence of others and in a group), and individual (gender, age, speed, and waiting patience).
- The literature should highlight these aspects as well, which will add more value to the current study context.

**Method**

**Questionnaire**

 Participants completed a questionnaire of 20 questions about pedestrian behaviors by Deb et al. (2017).

- Are these questions about general pedestrian behavior (compliance) at signalized crosswalks?
- Whether the short version questionnaire set tested locally to optimize the time?
- What was the average completion time and response rate?
- As this is an online survey, does this represent the French population? If not, isn’t it introduce bias in the study results?
- Whether the sample size is enough?

**2.4 Statistical Analysis**

 The statistical analysis part is a little bit difficult to understand. It is recommended to add a flowchart of the entire method and describe the methodology systematically. This will provide a better understanding to the readers and help them reproduce the results.

- PCA and clustering techniques are common to identify internal relationships (similarities). The authors should explain the methods briefly to improve the understanding of the statistical analysis. 

**Results**

 The first cluster includes the flower, and a man and a woman with neutral expressions. It has a lower influence on the scores (see green elements in Table 1; participants do not feel surprised, scared, observed or inhibited).

- There are no green elements in Table 1. The color coding for the clusters is missing.

Table 1: Colors indicate the clusters assessed by the HCPC, from the least intense (green) to the most intense (red) feeling.

- The colors are not visible in Table 1. Fix the color coding in the table.

Due to missing color coding (green and red) in Table 1, it is impossible to validate some of the statements. The authors should fix the table.

**Discussion**

 No link was found between the results of our questionnaire and those of the Pedestrian Behavior Questionnaire (PBQ). We expected participants showing high scores for violations and aggressive behaviors in the PBQ (or low scores for positive behaviors) to feel less observed or scared by the eye images. However, no correlation was found.

- The online response and real-world scenario of crossing decisions are different. A crossing decision in a dangerous situation is not just born by what eye expression other gives when going against the social norm (like violating a signal). Still, it also depends on many factors (traffic, infrastructure, and situational). The author should explore and add these factors to explain the study outcomes.

**Conclusions**

 In a meta-analysis of 15 experiments from 13 research papers (Dear et al., 2019), found a 35% reduction in the risk of antisocial behavior when eye images are present. In contrast, systematic reviews have suggested that CCTV cameras reduce crime by only 16%.

- The example presented by the author is context specific. The presence of CCTV indeed reduces crime (in particular places). But jaywalking or signal violation is not considered a crime in many countries, especially in developing countries, where rules and regulations are not strict and there are no penalties for signal violation. Thus, just promoting the positive value of social control alone is not enough to enhance compliance; traffic and infrastructure-related safety measures are also needed. Authors should explore and add more transportation study examples that would help better explain the outcomes.
- An online questionnaire survey won’t reflect how pedestrians respond in real-world traffic situations. Past studies showed that other people's presence impacts signal compliance:
  - a) Rosenbloom, T., 2009. Crossing at a red light: Behaviour of individuals and groups. Transp. Res. Part F: Traffic Psychol. Behav. 12 (5), 389–394.
  - b) Faria, J.J., Krause, S., Krause, J., 2010. Collective behavior in road crossing pedestrians: the role of social information. Behav. Ecol. 21 (6), 1236–1242.
  - c) Raoniar, R. and Maurya, A. K., 2022. Pedestrian Red-Light Violation at Signalized Intersection Crosswalks: Influence of Social and Non-Social Factors, Safety Science, vol. 147. pp. 105583.

Seeing different emotions disseminated through eye expressions could reflect the emotional response from a peer or group member while someone in the group goes against the norm. But a person might not see others' facial reactions (non-peers) while making the final crossing decision. 

- Do facial expression/eye image alone enough to improve compliance? If not, why?
- Authors need to validate the findings in a controlled experimental setting or through a real-world observational setting. If not possible in the current study context, it should be highlighted in the study limitations.

Is the work clearly and accurately presented and does it cite the current literature?

Yes

If applicable, is the statistical analysis and its interpretation appropriate?

Yes

Are all the source data underlying the results available to ensure full reproducibility?

Yes

Is the study design appropriate and is the work technically sound?

Yes

Are the conclusions drawn adequately supported by the results?

Yes

Are sufficient details of methods and analysis provided to allow replication by others?

Partly

Reviewer Expertise:

Walkable and Cyclable Cities, Road Safety, Injury Prevention, Transport Psychology, Transportation Accessibility, and Quantitative Social Research.

I confirm that I have read this submission and believe that I have an appropriate level of expertise to confirm that it is of an acceptable scientific standard, however I have significant reservations, as outlined above.

## References

[1] : Crossing at a red light: Behaviour of individuals and groups. *Transportation Research Part F: Traffic Psychology and Behaviour* .2009;12(5) : 10.1016/j.trf.2009.05.002 389-394 10.1016/j.trf.2009.05.002

[2] : Collective behavior in road crossing pedestrians: the role of social information. *Behavioral Ecology* .2010;21(6) : 10.1093/beheco/arq141 1236-1242 10.1093/beheco/arq141

[3] : Pedestrian red-light violation at signalised intersection crosswalks: Influence of social and non-social factors. *Safety Science* .2022;147: 10.1016/j.ssci.2021.105583 10.1016/j.ssci.2021.105583

# Response

**Published:** 2023-06-26

**Authors:** Cédric Sueur

**Affiliations:** Université de Starsbourg, France

The study focuses on exploring the effect of eye image on pedestrian road crossing behavior (signal violation). The overall work is good; still, the authors need to address the following comments to strengthen the manuscript further:

**Abstract**

 None

**Introduction**

 Human eye contact, facial expressions (smile), and hand gestures could lead to changes in driver behavior (page 3 of the pdf) and also helps pedestrian to cross busy roads.

- These might not be the only factors that determine the signal violation behavior. There are numerous factors like non-social (traffic volume, composition, infrastructure) and social (presence of others and in a group), and individual (gender, age, speed, and waiting patience).
- The literature should highlight these aspects as well, which will add more value to the current study context.

**Response: **We added a new section in the introduction about all these factors.

**Method**

**Questionnaire**

 Participants completed a questionnaire of 20 questions about pedestrian behaviors by Deb et al. (2017).

- Are these questions about general pedestrian behavior (compliance) at signalized crosswalks?

**Response: **We indicated the information: “The Pedestrian Behaviour Scale (PBS) is a self-report questionnaire that distinguishes five dimensions of pedestrian behaviour. Most questions concern pedestrian crossing at signalised crosswalk but some not (Vandroux et al. 2022).”

- Whether the short version questionnaire set tested locally to optimize the time?

**Response:** Yes, this is indicated in the paper (This questionnaire was originally developed by Tom and Granié (2011) with 47 questions. We chose the short version, which is considered as reliable as the long version according to Deb et al. (2017) and Tom and Granié (2011), in order to avoid demotivating participants.) but we added details : “We used a pedestrian behavior questionnaire (PBQ) with 20 questions, developed by ( Deb et al., 2017) and taken from ( Tom and Granié, 2011) to optimise time as some original questions gave correlated answers (Vandroux et al. 2022).”

  

- What was the average completion time and response rate?

**Response: **The response rate was 42.15%. The average completion time was 334±310 sec. We added this information.

  

- As this is an online survey, does this represent the French population? If not, isn’t it introduce bias in the study results?

**Response: **Yes, this is indicated in the paper: “The survey was created using LimeSurvey ( Engard, 2009; Jayasundara et al., 2010; LimeSurvey Project Team, 2012) and administered online to the French population through mails and social media (Facebook and Twitter).”

  

- Whether the sample size is enough?

**Response: **Yes, we indicated the information: As per power computation of sample size using G*Power version 3.1.9.7, reported 262 participants were required for this research at an α value 0.05, power equal to 0.95 and with effect size 0.20.

**2.4 Statistical Analysis**

 The statistical analysis part is a little bit difficult to understand. It is recommended to add a flowchart of the entire method and describe the methodology systematically. This will provide a better understanding to the readers and help them reproduce the results.

 Response: We added an introduction sentence and some bullet points to make the understanding easier.

- PCA and clustering techniques are common to identify internal relationships (similarities). The authors should explain the methods briefly to improve the understanding of the statistical analysis. 

**Response:** We added a paragraph on PCA and HCPC: PCA is a statistical method of reduction of variables in new dimensions usually meaningful. Variables are automatically corrected to be comparable (mean and range). Dimensions (groups of different variables) with eigenvalue superior to 1 are kept. We examined loadings of each variable on each dimension. The loadings are interpreted as the coefficients of the linear combination of the initial variables from which the principal components are constructed. The loadings are equal to the coordinates of the variables divided by the square root of the eigenvalue associated with the component. The goal of HCPC is to identify groups (i.e. clusters) of similar objects within a data set of interest.

**Results**

 The first cluster includes the flower, and a man and a woman with neutral expressions. It has a lower influence on the scores (see green elements in Table 1; participants do not feel surprised, scared, observed or inhibited).

- There are no green elements in Table 1. The color coding for the clusters is missing.

**Response : **sorry about that, this is not our fault but an editing error. This is corrected.

 Table 1: Colors indicate the clusters assessed by the HCPC, from the least intense (green) to the most intense (red) feeling.

- The colors are not visible in Table 1. Fix the color coding in the table.

**Response : **sorry about that, this is not our fault but an editing error. This is corrected.

 Due to missing color coding (green and red) in Table 1, it is impossible to validate some of the statements. The authors should fix the table.

**Response :** sorry about that, this is not our fault but an editing error. This is corrected.

**Discussion**

 No link was found between the results of our questionnaire and those of the Pedestrian Behavior Questionnaire (PBQ). We expected participants showing high scores for violations and aggressive behaviors in the PBQ (or low scores for positive behaviors) to feel less observed or scared by the eye images. However, no correlation was found.

- The online response and real-world scenario of crossing decisions are different. A crossing decision in a dangerous situation is not just born by what eye expression other gives when going against the social norm (like violating a signal). Still, it also depends on many factors (traffic, infrastructure, and situational). The author should explore and add these factors to explain the study outcomes.

**Response: **We agreed, but here we based this point on only psychological/intrinsic factors as both the PBQ questionnaire and the eye image questionnaire were made online. So we still think that participants showing high scores for violations and aggressive behaviors in the PBQ (or low scores for positive behaviors) should feel less observed or scared by the eye images. However, we added the other factors influencing the pedestrian behaviours in the introduction.

**Conclusions**

 In a meta-analysis of 15 experiments from 13 research papers (Dear et al., 2019), found a 35% reduction in the risk of antisocial behavior when eye images are present. In contrast, systematic reviews have suggested that CCTV cameras reduce crime by only 16%.

- The example presented by the author is context specific. The presence of CCTV indeed reduces crime (in particular places). But jaywalking or signal violation is not considered a crime in many countries, especially in developing countries, where rules and regulations are not strict and there are no penalties for signal violation. Thus, just promoting the positive value of social control alone is not enough to enhance compliance; traffic and infrastructure-related safety measures are also needed. Authors should explore and add more transportation study examples that would help better explain the outcomes.

**Response: **You are right, we added your point in the discussion.

- An online questionnaire survey won’t reflect how pedestrians respond in real-world traffic situations. Past studies showed that other people's presence impacts signal compliance:
  - a) Rosenbloom, T., 2009. Crossing at a red light: Behaviour of individuals and groups. Transp. Res. Part F: Traffic Psychol. Behav. 12 (5), 389–394.
  - b) Faria, J.J., Krause, S., Krause, J., 2010. Collective behavior in road crossing pedestrians: the role of social information. Behav. Ecol. 21 (6), 1236–1242.
  - c) Raoniar, R. and Maurya, A. K., 2022. Pedestrian Red-Light Violation at Signalized Intersection Crosswalks: Influence of Social and Non-Social Factors, Safety Science, vol. 147. pp. 105583.

**Response: **We added this element and references in the introduction.

 Seeing different emotions disseminated through eye expressions could reflect the emotional response from a peer or group member while someone in the group goes against the norm. But a person might not see others' facial reactions (non-peers) while making the final crossing decision. 

**Response: **We added this limitation of our study in the conclusion.

- Do facial expression/eye image alone enough to improve compliance? If not, why?

**Response: **We based our experiment on previous studies showing the importance of eyes in social control. Specifically, in sad and angry facial expressions, the eyes received more attention than the mouth (Eisenbarth and Alpers, 2011). It is right that different studies show also the importance of the mouth in facial expression recognition (Calvo and Fernández-Martín, 2013) but eyes tend to attract the highest proportion of fixations (Guo and Shaw, 2015). However, we wanted to build a protocol comparable to eye effect experiments as indicated in our introduction: “With this in mind, Bateson et al. (2006) conducted an experiment to test the effect of eye images on cooperative behavior. In the coffee area of a building at the University of Newcastle, an honesty box was used for people to make contributions to the coffee fund. The experiment consisted of placing pictures of eyes or flowers close to this box and assessing whether they led to differences in the contributions made. The authors found that in the presence of eye images, subjects paid on average 2.76 times more than when flowers images were displayed. Still comparing the effect of these flowers images as control to those of eye images, other studies have found similar prosocial effects during everyday events. For example, eye images had prosocial effects such as cooperation for the clearing of trays in a university cafeteria ( Ernest-Jones et al., 2011) and for waste sorting at a bus stop ( Francey and Bergmüller, 2012). Similar results were found in experiments in more specific contexts such as blood donations: While eye images on flyers did not result in differences in pledge with a logo as control, more “real” donations were made by students who got the flyers with eyes image ( Sénémeaud et al., 2017). Other than the activation of a “sense of being seen” ( Pfattheicher and Keller, 2015) or the desire to maintain a pro-social reputation ( Bateson et al., 2006), it is important to note that humans possess neurons that respond to faces and eyes and activate such prosocial behaviors ( Emery, 2000; Haxby et al., 2000). Bateson et al. (2013) suggest that eye images induce more pro-social behavior regardless of local norms, thus suggesting that the application of eye images could be a means to combat anti-social behavior by triggering a feeling of shame ( Nugier et al., 2007).”

 So eye effect is well showed in the literature and we obtained in our study the results we expected. However, we added a part in the discussion: “This might be due to the fact that eyes are not enough to convey a facial expression and the presence of the mouth on the face pictures could conduct to stronger effects than the ones we obtained (Calvo and Fernández-Martín, 2013; Eisenbarth and Alpers, 2011; Guo and Shaw, 2015)”

  

- Authors need to validate the findings in a controlled experimental setting or through a real-world observational setting. If not possible in the current study context, it should be highlighted in the study limitations.

**Response: **We added sentences in the conclusion to discuss this point. Indeed, we performed a controlled experiment in real-world situations and the paper is submitted. However this current study is still interesting as it allows to get sociodemographic information and a wide spectrum of people and cities that we cannot get in an empirical study.

# Reviewer report: Reviewer response for version 1

**DOI:** 10.5256/f1000research.80016.r167907

**Published:** 2023-04-17

**Authors:** Yunchang Zhang

**Affiliations:** 1 Desay SV, Singapore, Singapore

This is a well-established paper, and I enjoyed reading this paper. Several comments:

1. For the design of experiment, I’m wondering whether eye contact is enough to explain the feeling of friendly, neutral, surprise, anger, etc. I think more rigorous design or evidence is needed here to prove your control and treatment.  
2. I felt that a power analysis is missing in this paper, although fruitful statistical analyses have been conducted. A power analysis will be helpful to demonstrate you have enough samples for the following analyses.
3. Section 3.3, the authors should list the procedures of Kaiser-Meyer-Olkin measure and Bartlett’s test procedures and explain them in detail in this section. For example, a question will be why does a 0.80 of Kaiser-Meyer-Olkin measure is considered satisfactory?
4. When I read Tables 2 and 3, I got lost by the meanings of variables. What are the values of these variables? Indicators or continuous variables? I recommend using a table for these explanatory variables.
5. Additionally, the authors should state the number of comparisons in the Tukey multiple comparisons and the confidence level you choose to make the adjustments since 2 comparisons and 20 comparisons have significant differences.
6. The discussion of sociodemographic factors on behavior is vague. Neither did the authors provide statistical numbers, nor persuasive explanations about the differences between South of France and the rest of France.
7. I understand that there is limited literature regarding the impact of eye contact on pedestrian behavior, but I expected a well-established paragraph regarding the future directions of your research. For example, is a questionnaire adequate for the study? Would a simulator study or site observations be better etc?

Is the work clearly and accurately presented and does it cite the current literature?

Yes

If applicable, is the statistical analysis and its interpretation appropriate?

Yes

Are all the source data underlying the results available to ensure full reproducibility?

Partly

Is the study design appropriate and is the work technically sound?

Yes

Are the conclusions drawn adequately supported by the results?

Partly

Are sufficient details of methods and analysis provided to allow replication by others?

Yes

Reviewer Expertise:

Social Behavior, human interactions.

I confirm that I have read this submission and believe that I have an appropriate level of expertise to confirm that it is of an acceptable scientific standard, however I have significant reservations, as outlined above.

# Response

**Published:** 2023-06-26

**Authors:** Cédric Sueur

**Affiliations:** Université de Starsbourg, France

This is a well-established paper, and I enjoyed reading this paper. Several comments:

1. For the design of experiment, I’m wondering whether eye contact is enough to explain the feeling of friendly, neutral, surprise, anger, etc. I think more rigorous design or evidence is needed here to prove your control and treatment.  

**Response:** We based our experiment on previous studies showing the importance of eyes in social control. Specifically, in sad and angry facial expressions, the eyes received more attention than the mouth (Eisenbarth and Alpers, 2011). It is right that different studies show also the importance of the mouth in facial expression recognition (Calvo and Fernández-Martín, 2013) but eyes tend to attract the highest proportion of fixations (Guo and Shaw, 2015). However, we wanted to build a protocol comparable to eye effect experiments as indicated in our introduction: “With this in mind, Bateson et al. (2006) conducted an experiment to test the effect of eye images on cooperative behavior. In the coffee area of a building at the University of Newcastle, an honesty box was used for people to make contributions to the coffee fund. The experiment consisted of placing pictures of eyes or flowers close to this box and assessing whether they led to differences in the contributions made. The authors found that in the presence of eye images, subjects paid on average 2.76 times more than when flowers images were displayed. Still comparing the effect of these flowers images as control to those of eye images, other studies have found similar prosocial effects during everyday events. For example, eye images had prosocial effects such as cooperation for the clearing of trays in a university cafeteria ( Ernest-Jones et al., 2011) and for waste sorting at a bus stop ( Francey and Bergmüller, 2012). Similar results were found in experiments in more specific contexts such as blood donations: While eye images on flyers did not result in differences in pledge with a logo as control, more “real” donations were made by students who got the flyers with eyes image ( Sénémeaud et al., 2017). Other than the activation of a “sense of being seen” ( Pfattheicher and Keller, 2015) or the desire to maintain a pro-social reputation ( Bateson et al., 2006), it is important to note that humans possess neurons that respond to faces and eyes and activate such prosocial behaviors ( Emery, 2000; Haxby et al., 2000). Bateson et al. (2013) suggest that eye images induce more pro-social behavior regardless of local norms, thus suggesting that the application of eye images could be a means to combat anti-social behavior by triggering a feeling of shame ( Nugier et al., 2007).”

 So eye effect is well showed in the literature and we obtained in our study the results we expected. However, we added a part in the discussion: “This might be due to the fact that eyes are not enough to convey a facial expression and the presence of the mouth on the face pictures could conduct to stronger effects than the ones we obtained (Calvo and Fernández-Martín, 2013; Eisenbarth and Alpers, 2011; Guo and Shaw, 2015)”

  

1. I felt that a power analysis is missing in this paper, although fruitful statistical analyses have been conducted. A power analysis will be helpful to demonstrate you have enough samples for the following analyses.

**Response:** We added the power analysis: As per power computation of sample size using G*Power version3.1.9.7, reported 262 participants were required for this research at an α value 0.05, power equal to 0.95 and with effect size 0.20.

  

1. Section 3.3, the authors should list the procedures of Kaiser-Meyer-Olkin measure and Bartlett’s test procedures and explain them in detail in this section. For example, a question will be why does a 0.80 of Kaiser-Meyer-Olkin measure is considered satisfactory?

**Response: **We indicated more details about this part: “The results explain 49.7% of total variance. The Kaiser-Meyer-Olkin measure of sampling adequacy (indicating the proportion of variance in your variables that might be caused by underlying factors with high values, close to 1.0, generally indicate that a factor analysis may be useful with your data, Trujillo-Ortiz et al., 2006) was satisfactory and good (0.80) and the Bartlett’s test of sphericity was significant (p < 0.0001) indicating a homogeneity of variances.”

  

1. When I read Tables 2 and 3, I got lost by the meanings of variables. What are the values of these variables? Indicators or continuous variables? I recommend using a table for these explanatory variables.

**Response: **We indicated these details in the statistical analyses: “We assessed whether this score is influenced by the following dependent variables: the age (continuous variable), gender (factor: Male or Female), geographical zone of their city (factor with seven zones) or the city population size (as a factor categorized according to quartiles).”

 We added details about the values of these variables: “The questionnaire was received by 1,447 participants, 610 of whom answered the entire questionnaire (all three steps). The resulting dataset was used in our analyses (N=610). The mean time to answer the entire questionnaire was seven minutes. 71% of participants were women, and the mean age was 35 ± 14 years (min = 16, max = 84). The geographic repartition of the population is as follows: Hauts-de-France (N=17), Ile-de-France (N=100), Ouest (N=92), Est (N=219), Sud-Ouest (N=18), Sud-Est (N=75), Sud (N=89). Population size of cities was 237535±252149 (min = 46; max = 2220445). These factors were included in statistical analyses to avoid selection biases selection.” 

1. Additionally, the authors should state the number of comparisons in the Tukey multiple comparisons and the confidence level you choose to make the adjustments since 2 comparisons and 20 comparisons have significant differences.

**Response: **The Tukey multiple comparisons were made on the seven zones. We indicated the details in the statistical analyses: “For the “zone” variable (n = 7), an Anova followed by a Tukey posthoc test (df = 6, 603, confidence level = 0.95) was performed on the GLM residuals.” 

1. The discussion of sociodemographic factors on behavior is vague. Neither did the authors provide statistical numbers, nor persuasive explanations about the differences between South of France and the rest of France.

**Response:** We have looked for explanations about high violence rate in France but it is difficult to find some, first because it is forbidden in France to make link between sociodemographic factors, religion and criminality. However, we added sentences: Indeed, studies from the French Homeland Ministry reported that South of France is one of the regions with the highest violence rate. This region is also one with the highest road accident rate, mostly due to the population density (ONISR, 2021). However, reported per inhabitant, South of France, with Ile-De-France are the two regions with the highest contravention rate (ONISR, 2021).

  

1. I understand that there is limited literature regarding the impact of eye contact on pedestrian behavior, but I expected a well-established paragraph regarding the future directions of your research. For example, is a questionnaire adequate for the study? Would a simulator study or site observations be better etc?

**Response: **We added sentences about this in the conclusion. We realised a real-world situation experiment and submitted a paper. Moreover, we are working on a simulator study.

 References

 Calvo, M.G., Fernández-Martín, A., 2013. Can the eyes reveal a person’s emotions? Biasing role of the mouth expression. Motiv Emot 37, 202–211. https://doi.org/10.1007/s11031-012-9298-1

 Eisenbarth, H., Alpers, G.W., 2011. Happy mouth and sad eyes: Scanning emotional facial expressions. Emotion 11, 860–865. https://doi.org/10.1037/a0022758

 Guo, K., Shaw, H., 2015. Face in profile view reduces perceived facial expression intensity: An eye-tracking study. Acta Psychologica 155, 19–28. https://doi.org/10.1016/j.actpsy.2014.12.001

 Trujillo-Ortiz, A., Hernandez-Walls, R., Castro-Perez, A., Barba-Rojo, K., Otero-Limon, A., 2006. kmo: Kaiser-Meyer-Olkin Measure of Sampling Adequacy. A MATLAB file.[WWW document]. URL http://www. mathworks. com/matlabcentral/fileexchange/loadFile. do.
